# Ten Years After Bariatric Surgery: Bad Quality of Life Promotes the Need of Psychological Interventions

**DOI:** 10.3389/fpsyg.2018.02282

**Published:** 2018-11-22

**Authors:** Federica Galli, Marco Cavicchioli, Elena Vegni, Valerio Panizzo, Alessandro Giovanelli, Antonio Ettore Pontiroli, Giancarlo Micheletto

**Affiliations:** ^1^Department of Health Sciences, Università degli Studi di Milano, Milan, Italy; ^2^Department of Psychology, Vita-Salute San Raffaele University, Milan, Italy; ^3^U.O. Chirurgia Generale, Istituto Nazionale per la Chirurgia dell’Obesità, Istituto Clinico Sant’Ambrogio, Milan, Italy; ^4^U.O. Chirurgia Bariatrica, Istituto Nazionale per la Chirurgia dell’Obesità, Istituto Clinico Sant’Ambrogio, Milan, Italy; ^5^Department of Pathophysiology and Transplantation, Università degli Studi di Milano, Milan, Italy

**Keywords:** biliointestinal bypass, quality of life, follow-up, outcome, bariatric surgery, psychological interventions

## Abstract

**Background:** This study aims to evaluate long-term quality of life (QoL) and primary clinical outcomes, 10 years after biliointestinal bypass (BIB) surgery. It was expected that, although BIB might show encouraging primary outcomes, long term QoL could be significantly impaired.

**Methods:** Ninety patients were contacted for a phone interview [age 41.0 ± 10.6 (mean ± SD) years, age-range 31-65 years]. QoL (by SF-36) and the clinical situation (by *ad hoc* questionnaire) were collected. Data were analyzed with SPSS 22. SF-36 scores were compared with Italian normative data from general and healthy population. We also compared primary clinical outcomes and SF-36 scores between patients who reported high and low levels of satisfaction with BIB.

**Results:** Considering SF-36 results, patients showed significant impairments in QoL compared to general and healthy populations. Sixty-five percent would repeat the BIB. All patients showed at least one chronic adverse event. It occurred a significant decrease in pre-post co-occurrence rates of diabetes (χ^2^ = 18.41; *p* < 0.001) and hypertension (χ^2^ = 50.27; *p* < 0.001). Large and significant weight loss indexes (i.e., percent excess weight loss (%EWL); body mass index) were observed between pre-post intervention.

**Conclusion:** BIB showed promising primary clinical outcomes (i.e., hypertension, diabetes, and weight loss). However, subjects reported a significant impairment in all SF-36 domains. *Ad hoc* psychological interventions should be implemented to ameliorate the quality of life of these patients.

## Introduction

The obesity epidemic has reached alarming proportions in western countries. In the United States 34% of the population is obese (Flegal et al., 2010) and, in many European countries, including Spain, Italy, and United Kingdom, the prevalence of obesity is up to 20% (Berghöfer et al., 2008). With regard to bariatric interventions, biliointestinal bypass (BIB) is considered a purely malabsorptive procedure (Bressani Doldi et al., 1998; Micheletto et al., 2008). BIB has demonstrated a favorable risk-benefit relationship and positive metabolic effects, especially among patients with co-morbid obesity (BMI > 35) and type 2 diabetes, showing remission of diabetes in the majority of cases (Del Genio G et al., 2016).

However, although BIB and other bariatric surgery procedures have demonstrated relevant short-term effects considering primary clinical outcomes (i.e., weight loss and improvements of comorbid conditions), it is well-established that after the first year of intervention many patients start to gradually regain weight over several years (Karlsson et al., 2007), as well as it was observed how some psychological factors, such as alexithymia, are implicated in long-term weight regain (e.g., Lai et al., 2016; Paone et al., 2017). In addition to primary outcomes previously mentioned, several bariatric surgery studies considered quality of life (QoL) as a reliable outcome of surgical interventions efficacy (Hell et al., 2000; Nguyen et al., 2001; Nickel et al., 2017), especially considering a long-term follow-up period (Karlsson et al., 2007). QoL has been operationalized using *ad hoc* self-report questionnaires investing levels of satisfaction and functioning concerning emotional, physical, social, work, and romantic relationships domains (e.g., Moorehead-Ardelt QoL Questionnaire II; Moorehead et al., 2003). Specifically, the SF-36 (McHorney et al., 1993) is considered one of the gold standard instruments for assessing QoL among obese patients (Fontaine and Barofsky, 2001). Consistently, several studies revealed significant improvements of QoL in the first year after the intervention (e.g., Janik et al., 2016). Conversely, only few studies evaluated the QoL among bariatric patients over long periods and its relations with weight loss (Lindekilde et al., 2015; Mazer et al., 2017; Biron et al., 2018). A meta-analysis (Driscoll et al., 2016) on long-term follow-up (5–25 years) studies assessing QoL in bariatric patients found inconsistent results considering physical and mental health measures, outlining the importance of adding studies on this topic. Nevertheless, no studies have investigated such topic in BIB.

As a result, this study aims to evaluate the QoL in patients who underwent BIB procedure in a 10-year follow-up period. We decided to mainly focus on QoL, excluding other secondary outcomes, in light of its relevance for sustaining the implementation of psychological interventions among such clinical population (Fontaine and Barofsky, 2001). Primary clinical outcomes and subjective satisfaction are also considered in order to draw preliminary conclusions regarding the long-term efficacy of BIB procedure.

## Materials and Methods

This study was consistent with the code of conduct for research in psychology (Associazione Italiana per la Psicologia, 2015: Codice Etico per la Ricerca in Psicologia), in line with the ethical principles of the American Psychological Association [APA] (1992). All participants provided informed consent during the phone call, before the assessment interview. A phone call evaluation was performed to administer the SF-36 (Apolone and Mosconi, 1998; Apolone et al., 2005) in addition to a structured interview. The SF-36 consists of 36 questions enquiring about the general health status of patients, providing eight specific categories of physical and emotional scores (Physical Functioning, Role Physical, Bodily Pain, General Health, Vitality, Social Functioning, Role Emotional, and Mental Health) resumed on two main scales: Physical and Mental Composite Score. Very low rating for Physical score indicate severe physical dysfunction, distressful bodily pain, frequent tiredness, and poor evaluation of health. Very low rating for Mental score indicate psychological distress, severe social, and role disability due to emotional problems. We compared the scores of Italian healthy and general population (Apolone et al., 2005) with those of our sample of patients.

The structured interview detected the current BMI, onset age of obesity, current presence of diabetes and hypertension (self-reported), general satisfaction for the BIB, adverse events related to BIB. Expert clinical psychologist (FG) between September 2016 and March 2017 performed the phone interview. The phone call lasted about 45 min. From the medical records, we got data on BMI at baseline and 1-year post-intervention, hypertension and diabetes. During the phone call, it has been detected the subjective satisfaction with the surgery by asking “Would you repeat again the surgery?”, “Would you suggest the same intervention to a close friend?”. Only in the case of positive answers to the two questions the level of satisfaction was considered high (High Satisfaction Group-HSG), in the case of “no” answer(s) we considered the patient in the low level (Low Satisfaction Group-LSG). The protocol was approved by the Ethic Committee of S. Raffaele Hospital (CE 93/2016).

### Statistical Analysis

Cohen’s *d* and its 95% confidence interval (CI) (Borenstein et al., 2011) was computed in order to draw conclusions about the QoL of BIB patients after 10-year follow-up period. We decided to analyze data using such statistical procedure for several reasons. First of all, Cohen’s *d*, representing an effect size measure of standardized mean differences, informs the “practical significance” of study results (Cohen, 1992, 1994; Kirk, 1996; Vacha-Haase and Thompson, 2004). Secondly, procedures for computing Cohen’s *d* and its 95% CI are weighted for sample sizes. Eventually, ratio between Cohen’s *d* and its variance results in *Z*-value which is used to estimate the significance of standardized mean differences (Hedges and Olkin, 1985).

Considering clinical outcomes, χ^2^ and Φ index test were computed in order to compare the pre-post co-occurrence rates of diabetes and hypertension. Repeated measure ANCOVA (controlling for baseline BMI; age of patients; gender; years of follow-up) was utilized in order to demonstrate BIB efficacy in reducing BMI, considering three assessment phases: (a) pre-intervention; (b) post-intervention (12 months); and (c) 10 years after intervention. Eventually, independent *t*-test and Cohen’s *d* with its 95% CI were used to evaluate differences between intervention satisfaction groups (satisfied vs. not satisfied patients) considering BMI trend, percent excess weight loss (% EWL), occurrence of side effects, and SF-36 scores. In the light of missing data (8.8%) concerning post-surgery weight, we used expectation maximization method in order to impute such missing data. Missing completely at random assumption was demonstrated using test Little’s (1988) (χ^2^ (30) = 23.97; *p* = 0.77).

## Results

Two hundred and seventeen patients were contacted for the follow-up evaluation. Ninety subjects (41.5%; age 42.6 ± 9.71 years; 63 women and 27 men) were available for the phone interview; one patient declined the invitation to answer to the questionnaire; the other patients were unreachable by phone. The mean of years between intervention and current follow-up assessment was 10.31 ± 2.71, and ranged from 5 to 18 years. Obesity begun during infancy in 36 subjects (40%), adolescence in 19 (21%), and adulthood in 35 (39%) of patients.

Table 1 shows a detailed description of comparisons between obese patients and general/healthy populations considering SF-36 scores. Overall, we revealed significant and small to large differences in SF-36 scores comparing the previous groups. Particularly, 10 years after intervention, patients who underwent BIB procedure reported lower levels of QoL than general and healthy populations.

**Table 1:** Descriptive statistics and comparisons between bariatric patients (*N* = 90), general population (*N* = 2031), and healthy population (*N* = 608).

SF-36 dimensions	BP *M* (*SD*)	GP *M* (*SD*)	HP *M* (*SD*)	BP vs. GP *d* (95% CI)	BP vs. GP *Z*	BP vs. HP *d* (95% CI)	BP vs. HP *Z*
Physical functioning	77.55 (26.11)	84.46 (23.10)	97.30 (8.40)	-0.30 (-0.51 – 0.09)	-2.75^∗∗^	-1.62 (-1.85 – -1.38)	-13.38^∗∗∗^
Social functioning	68.33 (29.69)	77.43 (30.14)	86.40 (16.90)	-0.30 (-0.51 – -0.09)	-2.80^∗∗^	-0.95 (-1.18 – -0.72)	-8.20^∗∗∗^
Role limitations (physical problems)	66.38 (41.92)	78.21 (35.93)	94.30 (19.90)	-0.37 (-0.54 – -0.11)	-3.03^∗∗^	-1.17 (-1.40 – -0.94)	-9.98^∗∗∗^
Role limitations (emotional problems)	74.44 (36.05)	76.16 (37.25)	88.00 (28.40)	-0.05 (-0.26 – -0.16)	-0.43	-0.46 (-0.68 – -0.24)	-4.04^∗∗∗^
Pain	57.22 (32.36)	73.67 (27.65)	89.20 (18.20)	-0.59 (-0.80 – -0.38)	-5.46^∗∗∗^	-1.55 (-1.79 – -1.32)	-12.92^∗∗∗^
Mental health	64.53 (22.10)	66.59 (20.89)	75.8 (15.30)	-0.10 (-0.30 – -0.11)	-0.91	-0.69 (-0.91 – -0.46)	-6.03^∗∗∗^
Vitality	54.83 (21.11)	61.89 (20.69)	72.20 (15.40)	-0.34 (-0.55 – -0.13)	-3.16^∗∗∗^	-1.07 (-1.30 – -0.84)	-9.18^∗∗∗^
General health perception	53.44 (27.53)	65.22 (34.01)	80.20 (12.50)	-0.35 (-0.56 – -0.14)	-3.23^∗∗∗^	-1.75 (-1.99 – -1.51)	-14.33


*^∗∗^*p* < 0.01; ^∗∗∗^*p* < 0.001; BP, Bariatric patients; GP, General Population; HP, Healthy Population.*

Considering baseline primary clinical outcomes, subjects were affected by diabetes (14.4%) and hypertension (40%). After bariatric surgery, it was revealed significant decreases of co-occurrence rates of both diabetes (5.6%; χ^2^ = 18.41, *p* < 0.01; *Phi* = -0.45; *p <* 0.01) and hypertension (32.2%; χ^2^ = 50.27, *p* < 0.001; *Phi* = -0.74, *p* < 0.001). Independently of obesity severity, it was revealed a significant (*F*(1,88) = 13.26; *p* < 0.001) and large (*d* = 1.79;95% IC: 1.46–2.11) decrease of BMI between pre and post-surgical period. Non-significant (*F*(1,88) = 2.48; *p* = 0.12), albeit moderate (*d* = 0.61; 95% IC: 0.38–0.84) decrease between post-surgical and follow-up assessment was observed, as well as a significant (*F*(1,88) = 26.72; *p* < 0.001) and large (*d* = 2.40; 95%IC: 2.13–2.45) decrease between baseline and follow-up evaluation. Similar results were observed when we controlled the BMI trend for age of patients, gender and years of follow-up. Furthermore, the mean %EWL of patients was 45.44% (*SD* = 23.10) between the pre and post-surgical period and 59.63% (*SD* = 24.60) from the pre-surgical to the follow-up evaluation. All patients declared side effects linked to surgery (mean number: 3.0 ± 1.12): 73 (81.1%) reported flatulence, 57 (63.3%) proctological disorders, 44 (48.9%) polyarthralgia and 13 (14.4%) nephrolithiasis.

The 64.4% (*N* = 58) of subjects reported high levels of satisfaction with BIB procedure. Conversely, 35.6% (*N* = 32) of participants were characterized by low levels of satisfaction. These groups did not differ for age, gender, and comorbid conditions (diabetes and hypertension pre and post intervention). Table 2 summaries descriptive statistics and comparisons between these groups with regard to primary BIB clinical outcomes and SF-36 scores. Overall, patients who reported high levels of satisfaction showed better outcomes in terms of weight loss, number of side effects, and higher scores on some SF-36 dimensions, including physical functioning, vitality, and general health perception. Nevertheless, all patients reported the same impairments in social functioning and limitations in daily life related to physical and emotional problems.

**Table 2:** Study outcomes in relation to intervention satisfaction.

Outcomes	HSG (*N* = 58) *M*(*SD*)	LSG (*N* = 32) *M*(*SD*)	*t*_(88)_	*d* (95% CI)
BMI baseline	47.5 (7.50)	46.9 (6.73)	0.35	0.07 (-0.35 – 0.51)
BMI post-intervention	33.6 (5.64)	37.9 (6.40)	-3.32^**^	-0.73 (-1.17 – -0.29)
BMI 10-year follow-up	29.45 (4.00)	35.1 (6.80)	-4.32^***^	-1.10 (-1.56 – -0.64)
% EWL pre-post	53.08 (18.18)	31.48 (24.86)	4.50^***^	1.04 (0.58 – 1.50)
% EWL pre- follow-up	68.51 (14.60)	43.54 (30.52)	4.36^***^	1.16 (0.70 – 1.62)
Number of side effects	2.86 (1.13)	3.34 (1.03)	-1.99^*^	-0.44 (-0.87 – -0.002)
Physical functioning	84.13 (21.13)	65.62 (30.15)	3.08^**^	0.71 (0.26 – 1.15)
Social functioning	72.20 (28.96)	61.32 (30.18)	1.68	0.37 (0.06 –0.80)
Role limitations (physical problems)	72.84 (39.25)	54.68 (44.64)	1.92	0.44 (0.006 – 0.88)
Role limitations (emotional problems)	78.16 (32.17)	67.70 (41.89)	1.22	0.29 (-0.14 – 0.72)
Pain	62.93 (30.89)	46.87 (32.87)	2.30^*^	0.51 (0.07 – 0.95)
Mental health	68.62 (20.03)	57.12 (24.02)	2.42^*^	0.53 (0.10 – 0.97)
Vitality	59.39 (18.68)	46.56 (22.98)	2.87^**^	0.63 (0.19 – 1.07)
General health perception	60.77 (25.62)	40.15 (26.19)	3.62^***^	0.80 (0.35 – 1.24)


*^∗^*p <* 0.05; ^∗∗^*p* < 0.01; ^∗∗∗^*p* < 0.001. HSG, High Satisfaction Group; LSG, Low Satisfaction Group.*

## Discussion

Our follow-up study showed good results considering the traditional outcomes of bariatric surgery (weight loss and improvement of comorbid conditions), but less clear findings considering QoL and self-reported overall satisfaction.

Considering the traditional outcomes, BIB demonstrated efficacy in controlling diabetes and hypertension, showed elevated %EWL and a significant reduction of BMI, with 65% of patients who would repeat this bariatric procedure. The long-term remission rate of diabetes is in line with other follow-up studies (Brethauer et al., 2013; Schauer et al., 2017), that evidenced larger results compared to conventional medical therapies (Ribaric et al., 2014).

In spite of higher %EWL (59.63%) compared to other similar follow-up (16–27%) (Karlsson et al., 2007; Canetti et al., 2016), BIB patients showed an overall impaired QoL. Recent indications for surgery of obesity and weight-related diseases (Jumbe et al., 2016) state that in long-term follow-up after surgery the QoL follows the trend of body weight, and the QoL remains satisfactory if %EWL is maintained above 10%. Furthermore, even though HSG seemed to show a better QoL related to physical health than LSG, they manifested the same degree of impairment in daily activities as consequence of physical pain and emotional difficulties. Unfortunately, we do not have a QoL measure at the time of bariatric intervention. However, the photo we caught on the current situation require attention, because the QoL of these patients outlines important elements of frailty that we cannot ignore in any clinical setting. The social, emotional, and physical functioning of these patients showed critical points, that need to be better understood and clinically handled.

The psychological situation (mental health) of bariatric patients has been outlined to be subjected to an important decline in a long term follow-up (10 years) (Canetti et al., 2016), while it seems to be improved in a small time span (2 year or less follow-up) (Jumbe et al., 2016). After a period (about 1–2 years) of “honeymoon” following the bariatric procedure, for patients seem to begin a difficult phase to cope with. We think that such a group of patients (more than one third in our study would not repeat BIB) should be better studied to understand which mechanisms might be linked to the inadequate satisfaction with the intervention. By the psychological perspective much remain to be understood about this phase, and long-term follow-up are compelling.

Our data presents different conclusions: if we consider the subjective level of satisfaction with the intervention, we find an overall relationship with BMI decrease, but evaluating the same patients according to the QoL scoring the situation changes in the direction of a critical impairment in all domains of SF-36. This aspect opens to several considerations. It has been outlined that the QoL of patients treated with surgery is unrelated to the kind of bariatric procedure (De Luca et al., 2016), but rather it is related to the loss/regain of weight (Karlsson et al., 2007). In the case of our study, the %EWL remained significant over time, but all dimensions of SF-36 were significantly impaired when compared to general and healthy subjects. The impaired QoL has been linked to the deterioration of mental health found in another 10-year follow-up study (Canetti et al., 2016). Our data lead in other directions, because all domains of SF-36 were undermined in the same way as the domain of mental health. Impaired quality of life and worsening of mental health should be examined by further long-term studies.

Our study is not free from limitations. We collected data from a phone interview and cannot exclude that clinical data might be over or under-reported by patients. We did not have a control group with other kind of bariatric surgery to compare the QoL. Last, but not least, we do not have a measure of QoL at the time before intervention. Future directions for studies should address QoL dimensions in different bariatric procedures over a long period of time, in addition to BMI and clinical improvement.

## Conclusion

In front of a good clinical outcome, patients who underwent BIB showed significant impairments in all domains of QoL when compared with general and healthy population. This aspect leads to the importance of implementing psychological interventions for the patients, not only as an initial step following the intervention itself, but in a long time perspective.

## Author Contributions

FG concepted and designed the research; supervised the work; acquired the data; analyzed and interpreted the data; drafted the manuscript; and critically revised the manuscript. MC did the statistical analysis and analyzed and interpreted the data. EV concepted and designed the research and critically revised the manuscript. VP recruited the patients and critically revised the manuscript. AG recruited the patients. AP critically revised the manuscript. GM concepted and designed the research; supervised the work; and critically revised the manuscript.

## Conflict of Interest Statement

The authors declare that the research was conducted in the absence of any commercial or financial relationships that could be construed as a potential conflict of interest.
